# Isolation and Analysis of Donor Chromosomal Genes Whose Deficiency Is Responsible for Accelerating Bacterial and *Trans*-Kingdom Conjugations by IncP1 T4SS Machinery

**DOI:** 10.3389/fmicb.2021.620535

**Published:** 2021-05-20

**Authors:** Fatin Iffah Rasyiqah Mohamad Zoolkefli, Kazuki Moriguchi, Yunjae Cho, Kazuya Kiyokawa, Shinji Yamamoto, Katsunori Suzuki

**Affiliations:** ^1^Department of Biological Science, Graduate School of Science, Hiroshima University, Higashihiroshima, Japan; ^2^Program of Basic Biology, Graduate School of Integrated Sciences for Life, Hiroshima University, Higashihiroshima, Japan; ^3^Department of Biological Science, Faculty of Science, Hiroshima University, Higashihiroshima, Japan

**Keywords:** genome-wide screening, IncP1-type plasmid, *trans*-kingdom conjugation, type IV secretion system, horizontal gene transfer

## Abstract

Conjugal transfer is a major driving force of genetic exchange in eubacteria, and the system in IncP1-type broad-host-range plasmids transfers DNA even to eukaryotes and archaea in a process known as *trans*-kingdom conjugation (TKC). Although conjugation factors encoded on plasmids have been extensively analyzed, those on the donor chromosome have not. To identify the potential conjugation factor(s), a genome-wide survey on a comprehensive collection of *Escherichia coli* gene knockout mutants (Keio collection) as donors to *Saccharomyces cerevisiae* recipients was performed using a conjugal transfer system mediated by the type IV secretion system (T4SS) of the IncP1α plasmid. Out of 3,884 mutants, three mutants (Δ*frmR*, Δ*sufA*, and Δ*iscA*) were isolated, which showed an increase by one order of magnitude in both *E. coli*–*E. coli* and *E. coli*–yeast conjugations without an increase in the mRNA accumulation level for the conjugation related genes examined. The double-knockout mutants for these genes (Δ*frmR*Δ*sufA* and Δ*iscA*Δ*frmR*) did not show synergistic effects on the conjugation efficiency, suggesting that these factors affect a common step in the conjugation machinery. The three mutants demonstrated increased conjugation efficiency in IncP1β-type but not in IncN- and IncW-type broad-host-range plasmid transfers, and the homologous gene knockout mutants against the three genes in *Agrobacterium tumefaciens* also showed increased TKC efficiency. These results suggest the existence of a specific regulatory system in IncP1 plasmids that enables the control of conjugation efficiency in different hosts, which could be utilized for the development of donor strains as gene introduction tools into bacteria, eukaryotes, and archaea.

## Introduction

The conjugal transfer mechanism is a major driving force of genetic exchange between bacteria. Besides that, this mechanism can also occur from bacteria to either eukaryotes or archaea, known as TKC (Dodsworth et al., 2010; Moriguchi et al., 2013a; Garushyants et al., 2015; Lacroix and Citovsky, 2016). TKC is a type of horizontal gene transfer (HGT), which differs from vertical gene transfer (VGT), and promotes the transfer of genetic materials between non-related species. Within a prokaryotic population, HGT occurs ubiquitously and permits fast dissemination of new genes, which is essential for species adaptation and survival. This mechanism has been acknowledged as a driving force for the evolution of bacterial species (Richard et al., 2017; Ward et al., 2018).

Conjugation is a mechanism which involves the transfer of genetic material from donor to recipient cells due to the expression and regulation of their responsible genes harbored within the conjugative plasmid [e.g., IncP1 (Pansegrau et al., 1994; Haase et al., 1995), IncN (Winans and Walker, 1985; Yeo et al., 2003), and IncW (Fernández-López et al., 2006) plasmids] in the donor cells. In comparison to other incompatibility group plasmids including IncN and IncW plasmids, the IncP1-type plasmid has been suggested to have a broader host range, as it carries genomic signatures, which are predicted to be derived from various host origins (Suzuki et al., 2010). This IncP1-type plasmid has the ability to be transferred and replicated in hosts belonging to at least three proteobacteria subclasses: *Alphaproteobacteria* (Schmidhauser and Helinski, 1985; Yano et al., 2013), *Betaproteobacteria* (Kamachi et al., 2006; Suzuki et al., 2010), and *Gammaproteobacteria* (Schmidhauser and Helinski, 1985; Adamczyk and Jagura-Burdzy, 2003; Suzuki et al., 2010; Norberg et al., 2011). The ability of this IncP1-type plasmid to be adapted to, and replicated in different hosts confer its potential as gene introduction tool.

In a previous study, the IncP1-type conjugation system was reported to give a detectable DNA transfer to yeast, in addition to proteobacteria (Hayman and Bolen, 1993). IncF1 and IncI1 conjugation systems showed undetectable DNA transfer to yeast, although comparable DNA transfer to proteobacteria as IncP1-type system was observed (Bates et al., 1998). This broader transferability of the IncP1-type plasmid, such as to yeast, employed the usage of this plasmid as a gene introduction tool. Generally, to make convenient to use as the tool, a native self-transmissible plasmid (e.g., RP4 plasmid) is separated into two parts, a helper plasmid and a shuttle vector. The helper plasmid provides genes for the biosynthesis of the conjugative pilus and production of stable mating aggregates (IncP1-T4SS) for the transfer, and genes for mobilization. On the other hand, the shuttle vector comprises of origin of transfer (*oriT*) derived from the IncP1 plasmid and genes for the plasmid maintenance and propagation within the donor and recipient during the conjugation process. Besides the IncP1-type shuttle vectors, an IncQ-type mobilizable plasmid is alternatively used as a backbone of the shuttle vector, as it is also transferred to the recipient cells, facilitated by helper plasmids derived from IncP1α (Moriguchi et al., 2013b) or IncP1β (Willetts and Crowther, 1981; Moriguchi et al., 2016) subfamilies such as RP4 and R751 plasmids, respectively. This shuttle vector-and-helper system has been used by researchers as gene introduction tool from proteobacteria to various recipient organisms, such as, yeast (Moriguchi et al., 2013a,b; Soltysiak et al., 2019), archaea (Dodsworth et al., 2010), diatoms (Karas et al., 2015), and plant (Regnard et al., 2010).

Recently, publications related to the identification of chromosomal gene(s) within the donor cells that is responsible in promoting HGT of the RP4 plasmid upon the abiotic stress exposures (e.g., antibiotics or heavy metals exposures) have been reported. These studies performed genome-wide expression analysis of the chromosomal genes by using transcriptome analysis in relation to the expression of the selected conjugal-transfer genes in RP4 plasmid and/or genes that are probably responsible for the physiological changes of the donor cell, consequently affecting the HGT (e.g., SOS or/and Reactive Oxygen Species-related genes) (Shun-Mei et al., 2018; Zhang et al., 2019). However, in these studies, the screening approach is based on the expression analysis of the various genes within the stress-exposed donor cells which possibly influence the conjugation mechanism, and no functionality test was performed on the isolated candidate gene(s) for further validation. Thus, it is still not clear whether the genes expressed higher or less are correlated directly to the conjugation mechanism or not.

In our study, we identified and characterized the genetic features of the factor(s) within the *E. coli* genome that may influence the conjugative transfer mediated by IncP1α-type plasmids from an *E. coli* single-knockout mutant donor library. We focused on the “up”-mutants that have the ability to accelerate conjugative transfer to both prokaryotes and eukaryotes as they could be potent donor strains applicable to gene introduction tools. The isolated mutants were characterized by examining the possible correlation with the expression of the conjugation-related genes in the IncP1α plasmid. In addition, the generality of the improved characteristics by up-mutants was further characterized by assessing the conjugation efficiency of other broad-host-range plasmids, such as IncP1β, IncN, and IncW, as well as homologous gene mutants in another class of proteobacteria, *Agrobacterium tumefaciens* (Alphaproteobacteria).

## Materials and Methods

### Bacterial Strains, Yeast, and Growth Media

The bacterial strains and yeast used in this study are listed in Table 1. A complete set of *E. coli* non-essential gene deletion clones (Keio collection) was provided by the National BioResource Project (NBRP) of the Ministry of Education, Culture, Sports, Science and Technology (MEXT), Japan. All *E. coli* strains and *A. tumefaciens* were commonly cultured in LB Lennox medium at 37 and 28°C, respectively. In addition, *S. cerevisiae* was cultured in yeast-extract/peptone/dextrose (YPD) medium. Synthetic defined (SD) medium containing appropriate individual amino acids (leucine, 0.03 mg/mL; histidine, 0.02 mg/mL; and lysine, 0.03 mg/mL) was used as the selection media (SC–Ura) for yeast transconjugants at 28°C. Solid LB Lennox medium was prepared by the addition of 1.5% agar, and solid YPD and SC–Ura media were prepared by the addition of 2% agar. All components used in making these media, except polypeptone, were purchased from Becton, Dickinson, and Company (Franklin Lakes, NJ, United States) and supplied by Wako Pure Chemical Ind., Ltd. (Osaka, Japan). Appropriate antibiotics were added to the media at the following concentrations, which corresponded to the selection of bacteria and plasmids: ampicillin (Ap), 50 μg/mL; chloramphenicol (Cm), 30 μg/mL; gentamicin (Gm), 30 μg/mL; kanamycin (Km), 50 μg/mL; rifampicin (Rf), 30 μg/mL; streptomycin (Sm), 50 μg/mL; tetracycline (Tc), 7.5 μg/mL; and meropenem (Me), 10 μg/mL.

**TABLE 1:** Strains used in this study.

**Strains**	**Relevant characteristics**	**Source or reference**
***E. coli***		
Keio collection	An in-frame single-gene knockout mutant collection derived from BW25113, Km^R^	NBRP Japan
BW25113Δ*frmR*Δ*sufA*	*frmR* and *sufA* double-gene knockout mutant, constructed from Δ*frmR* derived from Keio collection, Km^R^	This study
BW25113Δ*iscA*Δ*frmR*	i*scA* and *frmR* double-gene knockout mutant, constructed from Δ*iscA* derived from Keio collection, Km^R^	This study
BW25113Δ*frmA*Δ*frmR*	*frmA* and *frmR* double-gene knockout mutant, constructed from Δ*frmA* derived from Keio collection, Km^R^	This study
BW25113Δ*frmB*Δ*frmR*	*frmB* and *frmR* double-gene knockout mutant, constructed from Δ*frmB* derived from Keio collection, Km^R^	This study
BW25113	*F*^–^Δ*(araD-araB)567 ΔlacZ4787(::rrnB-3) λ-rph-1 Δ(rhaD-rhaB)568 hsdR514*	NBRP Japan
SY327 (*λpir)*	*Δ(lac pro) argE(*Am*) recA56 λpir* Rif^R^ Nal^R^	NBRP Japan
S17-1 (*λpir)*	*F^–^ RP4-2(Km*^R^*::Tn7,Tc*^R^*::Mu-1) pro-82λpir recA1 endA1 thiE1 hsdR17 creC510*	NBRP Japan
***A. tumefaciens***		
C58C1	pTiC58-cured and Rif^R^ derivative of C58	Yamamoto et al., 2007
C58C1Δ*ATU_RS04380*	*ATU_RS04380* (*atu0890*) single-gene knockout derived from C58C1, Rif^R^	This study
C58C1Δ*ATU_RS08905*	*ATU_RS08905* (*atu1819*) single-gene knockout derived from C58C1, Rif^R^	This study
C58C1Δ*ATU_RS08390*	*ATU_RS08390* (*atu1713*) single-gene knockout derived from C58C1, Rif^R^	This study
***S. cerevisiae***		
BY4742	*MATα SSD1-V his3*Δ*1 leu2*Δ*0 lys2*Δ*0 ura3*Δ*0*	Invitrogen

### Construction of TKC *E. coli* Donor Library From Single-Knockout Keio Collection

The TKC *E. coli* donor library was constructed by introducing a helper plasmid, pRH220 (Nishikawa and Yoshida, 1998), and a TKC vector, pRS316:*oriT*^P^ (Moriguchi et al., 2013b), into the *E. coli* comprehensive gene knockout (KO) mutant collection [Keio collection (Baba et al., 2006)]. Both helper and vector plasmids were introduced into each KO mutant by conjugation as described in our previous report (Moriguchi et al., 2020). The conjugation reaction was performed under liquid conditions, followed by the selection of conjugants by inoculation to fresh selection LB Lennox medium containing Ap, Cm, and Km by 100-fold dilution at a 100 μL culture scale. The KO mutant lines that were successfully introduced with both helper and TKC vector were stored at −70°C as glycerol stocks. For the slow-growth mutant lines, which were anticipated to come from either slow growth, low conjugation efficiency, or antibiotic-susceptible phenotypes, the culture scale was increased up to 300 μL. The cultures were concentrated threefold before being stored as glycerol stocks. For the extremely slow-growth mutant lines, the conjugation reaction mixtures were directly spread onto solid selection plates and were incubated for up to 48 h at either 30°C or 37°C. Several colonies for each mutant were isolated and resuspended into the selection media, and then stored as glycerol stocks. In total, 3,884 mutant donor lines were constructed.

### Donor and Recipient Cell Cultures

The details of the genotypes for both donor and recipient cells as well as plasmids used in this study are described in Tables 1, 2, respectively. For the genome-wide screening analysis, donor *E. coli* Keio mutants and control BW25113 (pBBR122Δ*Cm*^R^) (IncP1α-pRH220, pRS316:*oriT*^P^) were inoculated from 96-well frozen stock plates using a 96-pinner tool and cultured in 100 μL medium supplemented with Ap, Cm, and Km in 96-well flat-bottom plates at 37°C for 15 to 18 h. For double-KO conjugation efficiency assessment, BW25113 single- and double-KO mutants and control (IncP1α-pRH220, pRS316:*oriT*^P^) were cultured in media supplemented with Ap and Cm. Details for the construction of *E. coli* double-knockout mutant strains are described in Supplementary Materials and Methods.

**TABLE 2:** Plasmids used in this study.

**Plasmids**	**Relevant characteristics**	**Source or References**
pK18mobsacB	Mobilizable plasmid; *sacB oriT Km^R^* used for the construction of *A. tumefaciens* knock-out mutant strains	Schäfer et al., 1994
pK18mobsacB-*ATU_RS04380*	Partial *ATU_RS04365, ATU_RS04370, ATU_RS04375, ATU_RS04380*, and *ATU_RS04385* integrated within pK18mobsacB; *Km*^R^	This study
pK18mobsacB-*ATU_RS08905*	Partial *ATU_RS08895, ATU_RS08900, ATU_RS08905, ATU_RS08910* and partial *nifS* integrated within pK18mobsacB; *Km*^R^	This study
pK18mobsacB-*ATU_RS08390*	*dgt*, *ATU_RS08390*, and partial *ATU_RS08395* integrated within pK18mobsacB; *Km*^R^	This study
pK18mobsacBΔ*ATU_RS04380*	*ATU_RS04380* single-gene knockout within pK18mobsacB-*ATU_RS04380*; *Km*^R^	This study
pK18mobsacBΔ*ATU_RS08905*	*ATU_RS08905* single-gene knockout within pK18mobsacB-*ATU_RS08905*; *Km*^R^	This study
pK18mobsacBΔ*ATU_RS08390*	*ATU_RS08390* single-gene knockout within pK18mobsacB-*ATU_RS08390*; *Km*^R^	This study
pJP5603sacBGmR	Mobilizable plasmid; *sacB oriT Gm*^R^ Used for the construction of *E. coli* complementation strains	This study *LC599391
pJP5603sacBGmR_*sufA*	Partial *menI*, *ydiH*, *RydB*, *sufA*, and *sufB* integrated within pJP5603sacBGmR	This study
pJP5603sacBGmR_*iscA*	*iscS, iscU, iscA, hscB*, and *hscA* integrated within pJP5603sacBGmR	This study
pJP5603sacBGmR_*frmR*	Partial *yaiX*, *yaiO, frmR, frmA*, and *frmB* integrated within pJP5603sacBGmR	This study
pBBR122Δ*Cm*^R^	Derivative of a commercially provided plasmid vector pBBR122; *Rep*^pBBR^’ (non-transmissible) *Km*^R^ Δ*Cm*^R^	Moriguchi et al., 2020
RP4	IncP1α-type conjugative broad host range plasmid; *Km*^R^ *Tc*^R^ *Ap*^R^	Pansegrau et al., 1994
pSa	IncW-type conjugative broad host range plasmid; *Cm*^R^ *Su*^R^ *Sp*^R^ *Sm*^R^ *Km*^R^ *Gm*^R^ *Tb*^R^	Tait et al., 1982
R46	IncN-type conjugative broad host range plasmid; *Tc*^R^ *Sm*^R^ *Su*^R^ *Ap*^R^	Brown and Willetts, 1981.
pRH220	Helper plasmid; *tra*^P1α^ *trb*^P1α^ *oriT*^P1α^ *ori*-pSC101 *Cm*^R^	*AB526840
pDPT51	Helper plasmid; *tra*^P1β^ *trb*^P1β^ *ori*-CoIE1 *Tp*^R^ *Ap*^R^	Taylor et al., 1983.
pRS316:*oriT*^P^	Mobilizable plasmid; *URA3 CEN6/ARSH4 ori*-pMB1 *Ap*^R^ *oriT*^RP4^	Moriguchi et al., 2013a.
pAY205	Mobilizable plasmid; *oriV*^Q^ *oriT*^Q^ *mob*^Q^ *URA3 TRP1 ARS1 Km*^R^ *Tc*^R^	*AB526841
pYN402	Mobilizable plasmid; *oriV*^Q^ *oriT*^Q^ *mob*^Q^ *URA3* 2 μ-*ori Gm*^R^	*AB531984

*DDBJ/EMBL/GenBank accession number.

Donor *E. coli* BW25113 Δ*sufA*, Δ*iscA*, or Δ*frmR* and control (IncW-pSa or IncN-R46) were cultured in media supplemented with Gm or Ap, respectively. Donor *E. coli* BW25113 Δ*sufA*, Δ*iscA*, or Δ*frmR* and control (IncP1β-pDPT51, pAY205) were cultured in media supplemented with Ap and Tc.

For the conjugation efficiency assessment in *Agrobacterium*, donor *A. tumefaciens* C58C1; Δ*ATU_RS04380*, Δ*ATU_RS08905*, or Δ*ATU_RS08390* (RP4, pYN402) corresponding to *frmR* as well as *sufA* and *iscA* in *E. coli*, respectively, were cultured in media supplemented with Gm, Km, and Rf at 28°C for 16 to 18 h. C58C1 was used as a control. Details for the construction of these strains are described in Supplementary Materials and Methods.

The recipient cells of the *S. cerevisiae* BY4742 strain or the *E. coli* SY327 strain were cultured in media supplemented without or with Rf, respectively, in 5 mL glass tubes for 18 to 22 h at 28°C or 16 to 18 h at 37°C, respectively, following the inoculation from the pre-cultured plates. Both donor and recipient cultures were pre-cultured with agitation to allow aeration.

### *Trans*-Kingdom Conjugation

Two screening strategies were performed in order to isolate the up-mutant candidates. The first screening was performed using rich medium (Supplementary Figure 1A), followed by the exclusion of antibiotic and nutrient reaction in the second screening (Supplementary Figure 2A) to create stringent conditions for transconjugant selection during the mating reaction. Fifty microliters of each donor overnight culture and 50 μL of yeast recipient suspension containing (2.0 × 10^6^ cfu/50 μL) were mixed in the first screening and was substituted from the cultured medium to TNB (80 mM Tris-HCl [pH 7.5], and 0.05% NaCl) in the second screening. Both donor and recipient were mixed and incubated at 28°C for 1 h, followed by the selection of transconjugants by spotting 15 μL of the conjugation reaction on SC–Ura supplemented with Tc. The culture plate was incubated for 48 to 72 h at 28°C. The TKC conjugation efficiency was calculated as the log_2_ value (number of transconjugants per median number of transconjugants of the control strain). The turbidity of the donor cells was measured using a microtiter-plate reader MTP-310 (Corona, Ibaraki, JAPAN).

For the standard TKC reaction, the suspension of donor *E. coli* strains in LB Lennox medium or *A. tumefaciens* C58C1 and recipient yeast in TNB containing 1.8 × 10^7^ or 5.0 × 10^7^ and 4.0 × 10^6^ cfu/300 μL of donors and recipient, respectively, were mixed. The donor overnight cultures of low living-cell ratio for *E. coli* double- and single-KO mutants (Δ*frmA* and Δ*frmB*) were concentrated to four-times their original concentration to adjust the number of living cells to an input cell number that was comparable to that of the wild-type control. The conjugation reaction was performed for up to 6 h for the assessment of IncP1α conjugation by the *E. coli* up-mutants. The conjugation reactions for the other TKC experiments were performed for 1 h. For the *A. tumefaciens* with yeast reaction, the scale of the reaction was increased to sevenfold to detect the transconjugant. TKC efficiency was determined based on the recovery of uracil prototrophic transconjugants supplemented with Tc or Me to inhibit the growth of donor *E. coli* and *A. tumefaciens*, respectively, calculated as the log_10_ value (number of transconjugants per recipient cell), and compared with the control.

### Bacterial Conjugation

We used a protocol identical to the one used for the TKC, where 300 μL of donor and SY327 recipient suspensions in LB Lennox medium were used during the conjugation reaction of up to 6 h co-cultivation for IncP1α conjugation assessment. The conjugation reaction for the other bacterial conjugation assessments was performed for 1 h. The conjugation reaction containing 1.8 × 10^7^ and 7.14 × 10^7^ cfu/300 μL of donor and recipient, respectively, was mixed. The transconjugants were selected on LB Lennox solid medium supplemented with Rf and the appropriate antibiotics for the selection of the transferred plasmid. Conjugation efficiency was calculated as the log_10_ value (number of transconjugants per recipient cell).

### Formaldehyde Treatment Assay

Prior to treating the donor cultures of Δ*frmR* and the control with formaldehyde, the overnight cultures of both donor and *E. coli* recipient were transferred into fresh LB Lennox medium (1:10 dilution) containing the appropriate antibiotics and then incubated at 37°C for 2 h with agitation. Then, 250 μM of formaldehyde was added to the donor cultures, and the incubation was continued for a further 2 h. For the non-treated controls, nothing was added to the donor cultures. After the incubation, donor (treated or non-treated with formaldehyde) and recipient cultures were subjected to conjugation using the procedure described in the bacterial conjugation methodology section (above).

### RNA Isolation and Quantitative RT-PCR

For the preparation of RNA, identical cultural conditions for *E. coli* donor cells as for the conjugation experiments were used. RNA was isolated using a NucleoSpin^®^ RNA kit, purchased from Macherey-Nagel GmbH & Co. KG (Dueren, Germany). For DNA removal and cDNA conversion, 2 μg of the total RNA was used as a template according to the manufacturer’s instructions using a PrimeScript^TM^ RT reagent kit with gDNA Eraser purchased from TaKaRa Bio Inc. (Shiga, Japan). The cDNA was subjected to RT-qPCR on a LightCycler^®^ 96 Instrument purchased from Roche Diagnostics Corporation (Indianapolis, IN, United States) using FastStart Essential DNA Green Master (Roche, Indianapolis, IN, United States). The expression levels of the target genes were normalized to the expression of the internal reference genes, *cysG* and *rrsA* (Zhou et al., 2011). All primers used in this experiment are listed in Supplementary Table 1.

### Statistical Analysis

Data were expressed as the mean ± standard error of the mean (SEM) of at least three independent biological experiments. The differences between groups were analyzed using Student’s *t*-test when two groups were compared by two-tailed, and one-way ANOVA (Tukey HSD analysis) for multiple group comparison. Analyses were performed using SPSS IBM Software for Windows, Version 17.0 (SPSS Inc., Chicago, IL, United States). Tests were considered statistically significant when *p* < 0.05, *p* < 0.01, or *p* < 0.001.

## Results

### Identification of High-TKC-Transfer *E. coli* Mutants by Genome-Wide Screening

In order to identify the mutants with high TKC ability, genome-wide screenings of donor *E. coli* single-KO gene mutations of Keio collection on plasmid transfer to yeast recipients were performed. Mutants that showed a log_2_ substituted relative TKC value equal to or greater than three (eightfold compared to parental strain) during the first screening were isolated prior to the second screening step. During this screening, the relative TKC efficiency of the mutant strains was normalized based on two different median value of parental control strains (median value of all control strains used in this screening, and median value of seven control strains in every experiment consisting of approximately 160 mutant strains) (Supplementary Figures 1B,C, respectively). Data of TKC efficiency in mutant strains using both normalizations were then integrated in order to avoid the exclusion of the possible up-mutant strains in every independent experiment. Mutant strains with log_2_ value equal to or greater than three in both or either of these two normalization methods were selected for the second screening. In total, 233 out of 3,884 mutants were isolated.

After performing the second screening (Supplementary Figure 2B), we selected the top three mutants out of the 233: Δ*frmR*, Δ*sufA*, and Δ*iscA*, which stably showed high TKC efficiency compared to the parental strain within triplicate experiments (sum log_2_ value ≥ 2.48). The *frmR* gene encodes FrmR transcriptional repressor protein on formaldehyde-sensing (*frm*) operon (Higgins and Giedroc, 2014; Denby et al., 2016), while the *sufA* and *iscA* genes encode the proteins within the iron–sulfur cluster assembly machinery (Lu et al., 2008).

### The Abilities of Both *E. coli*–*E. coli* and *E. coli*–Yeast Conjugations Were Improved in the Three Up-Mutants

**FIGURE 1:**
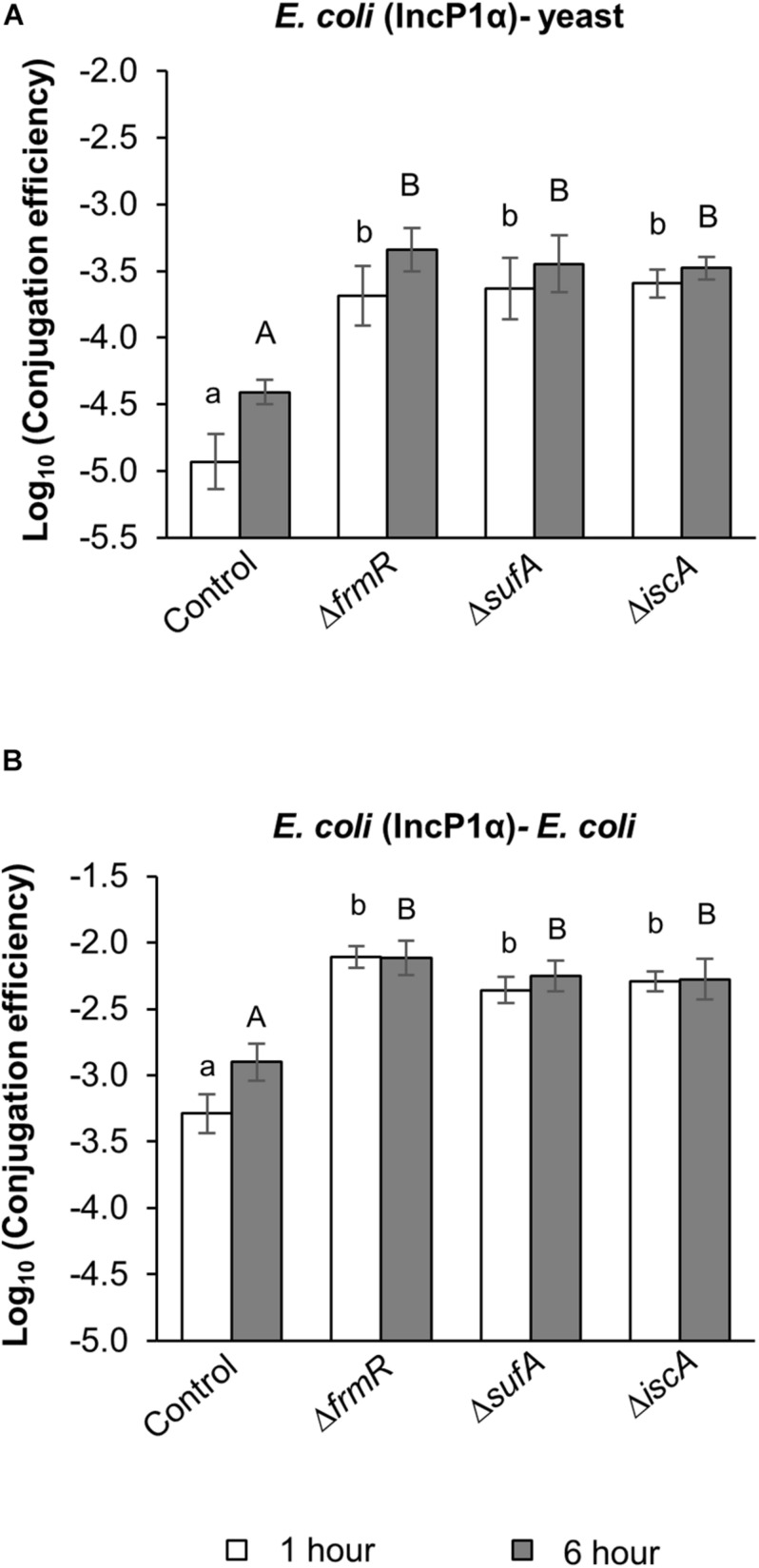
Effect of *frmR*, *sufA*, and *iscA* mutations in *Escherichia coli* on IncP1α conjugations. **(A)** TKC efficiency of IncP1α transfer from *E. coli* to yeast within four experimental replicates (*n* = 4). **(B)** Conjugation efficiency of IncP1α transfer from *E. coli* to *E. coli* within seven experimental replicates (*n* = 7). Both conjugation reactions were performed for 1 h (white bar) and 6 h (black bar). Data are presented as mean ± standard error of the mean (SEM). Different letters indicate significant differences between mutants and wild-type control at *p* < 0.05 using Tukey HSD multiple comparison analysis. BY4742 and SY327 were used as the recipients. BW25113 parental strain was used as the control.

The increases in TKC efficiency by Δ*frmR*, Δ*sufA*, and Δ*iscA* mutant donors compared to the parental strain were confirmed at different co-cultivation times (Figure 1A). At 1 h reaction, at least 17-fold increases in conjugation efficiency were observed in these mutants compared to the parental strain (Figure 1A). At 6 h reaction, at least ninefold increases in TKC efficiency were observed in these mutants compared to the parental strain.

In order to assess the effect of these mutations on the efficiency of bacterial conjugation, the corresponding conjugation reaction was performed with *E. coli* SY327 recipient cells. At 1 h co-cultivation, at least ninefold increases in conjugation efficiency were observed in these mutants compared to the parental strain (Figure 1B). At 6 h co-cultivation, at least fourfold increases in conjugation efficiency were observed in these mutants compared to the parental strain (Figure 1B). On the basis of these results, we conclude that these three up-mutants (Δ*frmR*, Δ*sufA*, and Δ*iscA*) increased *E. coli*–yeast TKC as well as *E. coli*–*E. coli* conjugation. The *E. coli*–yeast and *E. coli*–*E. coli* conjugation efficiencies of IncP1α between the up-mutants and the parental strain consistently showed significant difference at both 1 and 6 h co-cultivation (Figure 1). Thus, we integrated the conjugation reaction at 1 h co-cultivation after these experiments.

To confirm the repressing effect by the *frmR, sufA*, and *iscA* on the TKC of IncP1α plasmid, complementation analysis was performed by integrating the wild-type genes into the Δ*frmR*, Δ*sufA*, and Δ*iscA* donor mutant strains, respectively (Supplementary Figure 3). As a result, the repressing effect on TKC was restored within the complemented donor strains.

### Deficiency of *frmR*, *sufA*, and *iscA* Genes Can Affect Independently, but Not Synergistically to Activate IncP1α Plasmid Transfer

In order to assess the correlation between KO gene interaction and conjugation efficiency, double-KO mutant strains were constructed by introducing the second gene mutation, located within the same or different operons.

**FIGURE 2:**
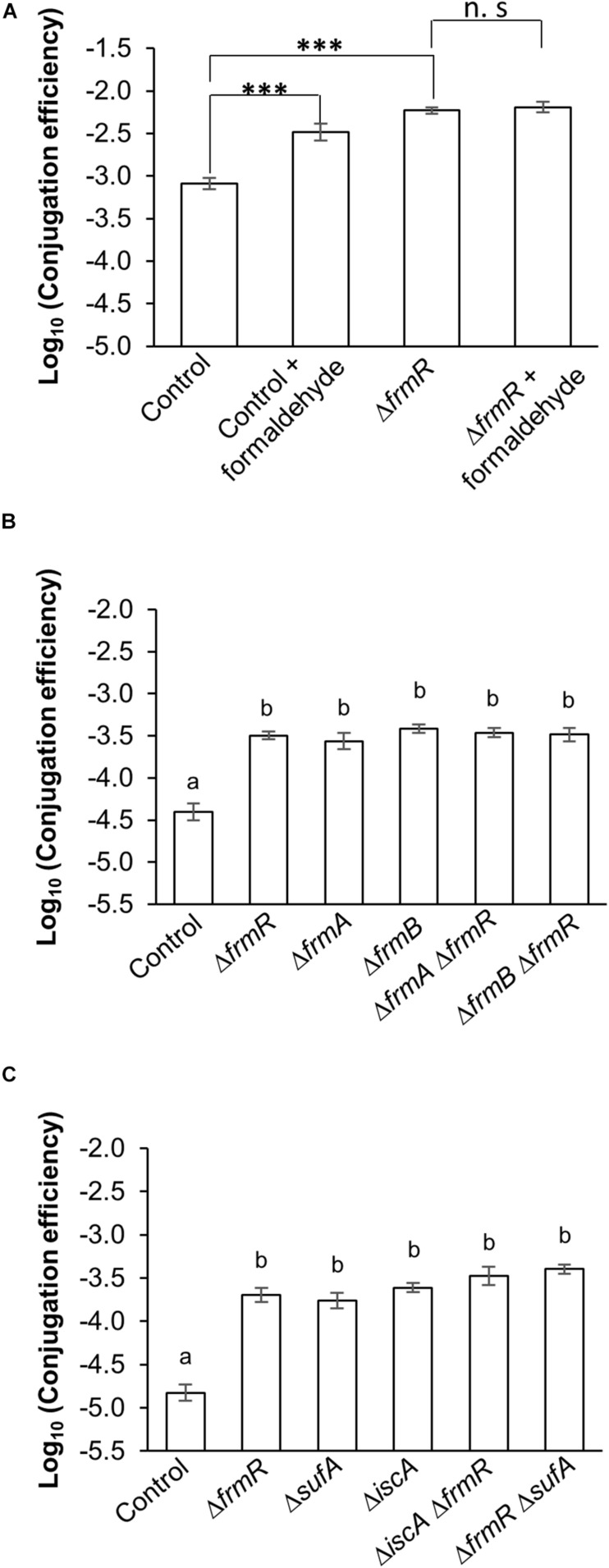
Confirmation analysis of the high conjugation efficiency in *frmR*, *sufA*, and *iscA* mutants. **(A)** Effect of formaldehyde (250 μM) on the conjugation efficiency of IncP1α plasmid transfer by Δ*frmR* mutants and wild-type control to *E. coli* recipient within five experimental replicates (*n* = 5). **(B)** TKC efficiency of IncP1α transfer by genes-deficient *E. coli* donor, belonging to the same operon (*frm* operon) within five experimental replicates (*n* = 5). **(C)** TKC efficiency of IncP1α transfer by genes-deficient *E. coli* donor, belonging to the different operons. This experiment was performed within 12 experimental replicates (*n* = 12) for single-KO mutants and wild-type control, while five experimental replicates were performed for double-KO mutants (*n* = 5). **(B,C)** BY4742 was used as the recipient. Data are presented as mean ± standard error of the mean (SEM). Asterisks (^∗∗∗^) indicate statistically significant difference at *p* < 0.001 (two-tailed *t*-test) compared to wild-type control. No significant difference is indicated as “n.s.” between treated and non-treated Δ*frmR* with formaldehyde. Different letters indicate significant differences between mutants and wild-type control at *p* < 0.05 using Tukey HSD multiple comparison analysis. BW25113 parental strain was used as the control. All conjugation reactions were performed for 1 h.

*frmR*, within the *frm* operon of *E. coli* K-12 derivatives, encodes a transcriptional repressor protein, FrmR (as a negative regulator), that specifically inactivates the expression of this operon in the absence of formaldehyde (Higgins and Giedroc, 2014; Denby et al., 2016; Osman et al., 2016). In the presence of formaldehyde, the expression of this operon is activated when the formaldehyde binds to the FrmR repressor, allowing induction of formaldehyde detoxification machinery catalyzed by FrmA and FrmB proteins, which are encoded by the downstream genes *frmA* and *frmB*, respectively (Denby et al., 2016; Osman et al., 2016). In this experiment, we hypothesized that the FrmR protein might be related to the increase in conjugation efficiency, due to its absence or inactivation, as the result of a deletion mutation or the binding of formaldehyde, respectively. In order to determine the effect of formaldehyde-dependent inactivation of FrmR repressor on the conjugation efficiency, both parental and Δ*frmR* donor strains were treated or non-treated with formaldehyde prior to the conjugation reaction. The effect on the conjugation efficiency of these treated strains were then compared with the non-treated strains. Based on the results obtained, an effect on conjugation efficiency of the parental strain with native *frmR* upon the addition of formaldehyde can be observed (about fourfold increase compared to the non-treated parental strain). In addition, no significant difference in conjugation efficiency between treated and non-treated Δ*frmR* with formaldehyde, although a significant increase in conjugation efficiency was observed (sevenfold) in comparison to the non-treated parental strain (Figure 2A). The result supported our hypothesis.

The TKC efficiency of the double-KO mutants (Δ*frmA*Δ*frmR* and Δ*frmB*Δ*frmR*) was not significantly different to those of single-KO mutants and not significantly different from each other but was significantly higher (at least sevenfold) compared to the parental strain (Figure 2B). This can possibly be attributed to the accumulation of endogenous ligands, including formaldehyde, which may inactivate the FrmR protein. This result indicates that neither *frmA* nor *frmB* alone directly affect the conjugation efficiency of IncP1α plasmids, so the effect is probably solely due to *frmR*.

The construction of the double-KO mutant, Δ*iscA*Δ*sufA*, was unsuccessful probably because of its synthetic lethality (Vinella et al., 2009). The KO of either of these genes with Δ*frmR* was constructed to discover the genetic interaction. It was observed that the TKC efficiency of the double-KO mutants, Δ*iscA*Δ*frmR* and Δ*frmR*Δ*sufA*, showed significant higher TKC efficiency compared to the parental strain by at least 11-fold, but showed no significant difference compared to the single-KO Δ*frmR*, Δ*sufA*, and Δ*iscA* (Figure 2C). These results indicate that the *frmR*, *sufA*, and *iscA* genes probably act on an identical step of the conjugation machinery of the IncP1α plasmid.

**FIGURE 3:**
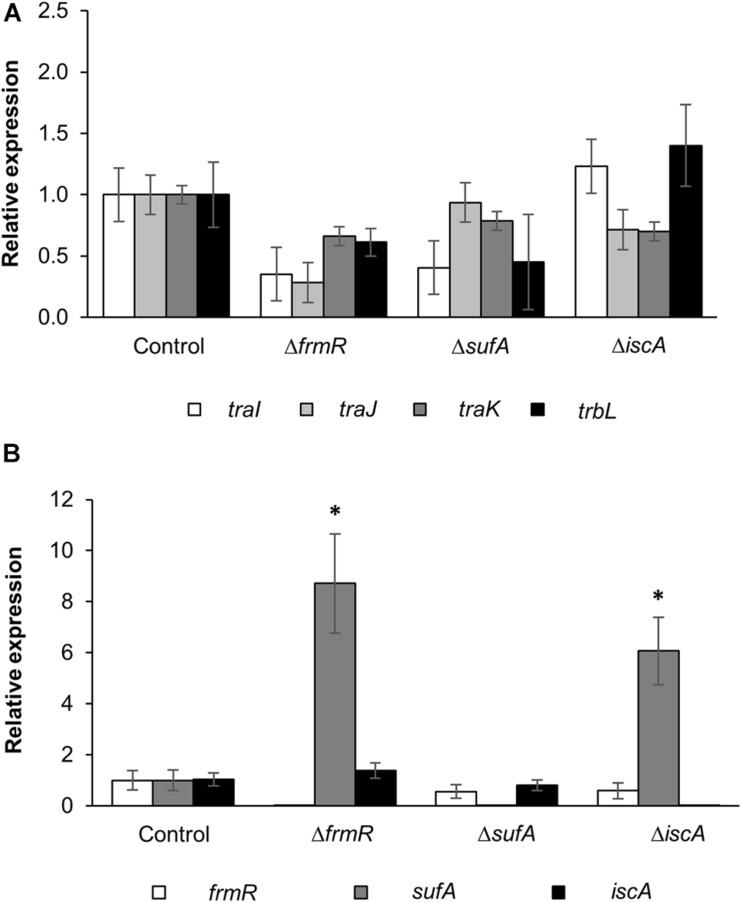
Expression analysis of conjugation-related and up-mutant genes by qRT-PCR. **(A)** Expression of *traI*, *traJ*, *traK*, and *trbL* genes within the helper plasmid, IncP1α-pRH220, harbored in the up-mutants and wild-type control donor strains, within triplicate experiments (*n* = 3). **(B)** Expression of *frmR*, *sufA*, and *iscA* genes within the up-mutant and wild-type control donor strains, within six experimental replicates (*n* = 6). Data are presented as mean ± standard error of the mean (SEM). Asterisk (^∗^) indicates statistically significant differences at *p* < 0.05 (two-tailed *t*-test) compared to wild-type control. BW25113 parental strain was used as the control.

In order to validate the correlation of the conjugation efficiency of these up-mutants with *tra* and *trb* genes expression, the basal expression levels of the selected *tra* and *trb* genes (*traI*, *traJ*, *traK*, and *trbL*) harbored within the pRH220 helper plasmid in the donor cells were compared between mutant and parental strains. These genes were selected as representatives of the three major operons within the RP4 IncP1α plasmid under the regulation of three major promoters: P_traJ_, P_traK_, and P_trbB_ (Pansegrau et al., 1994). As shown in Figure 3A, the expression of *traI*, *traJ*, *traK*, and *trbL* in the donor mutant strains of Δ*frmR*, Δ*sufA*, and Δ*iscA* showed no significant increase in comparison with the parental strain.

In order to assess the correlation between the basal gene expression of *sufA*, *iscA*, and *frmR* with conjugation efficiency, the expression of these genes within the up-mutant donor strains was evaluated (Figure 3B). Real-time PCR analysis revealed that *iscA* expression in both Δ*sufA* and Δ*frmR* mutants, as well as *frmR* expression in Δ*sufA* and Δ*iscA*, were not significantly different and comparable to the expression levels of the parental strain. These results indicate that *iscA* and *frmR* in the mutants expressed at the same level as in the *E. coli* parental strain. In addition, *sufA* gene expression in both Δ*iscA* and Δ*frmR* was significantly high (approximately six- and ninefold, respectively) compared to the parental strain. However, no complementary effect on the repression of conjugation efficiency in these two mutants was observed (Figure 2C). Therefore, no clear transcriptional interaction among the three genes, which links conjugation efficiency, was observed.

On the basis of the results from single- and double-KO mutant analyses, it may be suggested that defects of FrmR, SufA, and IscA can affect independently to terminate their repression of IncP1α plasmid conjugation, but probably act on the identical step(s) of conjugation machinery.

### The Enhancement of Conjugation Efficiency by Up-Mutants Specifically Affects IncP1-Type Plasmids Transfer

In order to observe the generality of the effect of these *E. coli* up-mutant strains on conjugation efficiency of the broad-host-range plasmids, the conjugation efficiency of IncN, IncW, and IncP1β to recipient cells was assessed and compared with that of the parental strain.

**FIGURE 4:**
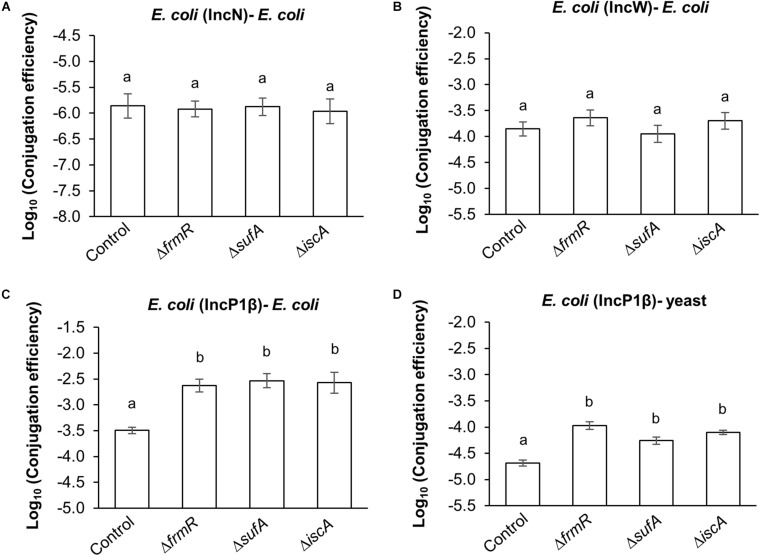
Generality assessment of *frmR*, *sufA*, and *iscA* mutations on the conjugation of broad host range plasmids. **(A)** Conjugation efficiency of IncN (R46) plasmid transfer to *E. coli* recipient cells within five experimental replicates (*n* = 5). **(B)** Conjugation efficiency of IncW (pSa) plasmid transfer to *E. coli* recipient cells within seven experimental replicates (*n* = 7). **(C)** Conjugation efficiency of IncP1β (pDPT51)-mediated shuttle vector (pAY205) transfer to *E. coli* and **(D)** yeast within triplicate experiments (*n* = 3). BY4742 and SY327 were used as the recipients. All conjugation reactions were performed for 1 h. Data are presented as mean ± standard error of the mean (SEM). Different letters indicate significant differences between mutants and wild-type control at *p* < 0.05 using Tukey HSD multiple comparison analysis. BW25113 parental strain was used as the control.

On the basis of the results obtained regarding the conjugation efficiency of IncN (R46) and IncW (pSa) to *E. coli* recipient cells, no significant difference was observed compared to the parental strain (Figures 4A,B). By contrast, an increase in conjugation efficiency was observed in both *E. coli* – *E. coli* and *E. coli* – yeast compared to the parental strain (seven–ninefold and three–fivefold, respectively), by measuring the transfer of an IncQ plasmid derived *E. coli*–yeast shuttle vector (pAY205) facilitated by IncP1β (pDPT51) (Figures 4C,D). No significant difference were observed among the up-mutant strains (Figures 4C,D). These results suggest that the enhancing effect of these mutants on conjugation efficiency in both prokaryotes and eukaryotes is probably specific to IncP1-type T4SS.

### Δ*ATU_RS04380*, Δ*ATU_RS08390*, and Δ*ATU_RS08905* Mutants for Agrobacteria Homologs of the Up-Mutant Genes Promote *Trans*-Kingdom Conjugation

In order to assess the generality effect of up-mutant genes on IncP1-type T4SS-mediated plasmid transfer in other bacterial species, TKC analysis was performed by combining an IncQ mobilizable plasmid (pYN402) transfer system mediated by an IncP1α-type plasmid (RP4) and mutants of *A. tumefaciens*.

The homologous *E. coli* up-mutant genes in *A. tumefaciens* strain C58 were selected based on BlastP score analysis, phylogenetic trees, and previously reported study (Higgins and Giedroc, 2014; Chen et al., 2016; Heindl et al., 2016). According to the BlastP analysis, the homologous gene of *frmR* (*ATU_RS04380*) had the highest similarity (46.03% amino acid identity and 2e–15 *e*-value). In addition, the shared homologous genes of *sufA* and *iscA*: *ATU_RS08390* and *ATU_RS08905* (*sufA*:37.14% and 43.69% amino acid identity; 6e–23 and 9e–26 *e*-value, respectively) as well as (*iscA*: 37.14% and 39.62% amino acid identity; 3e–23 and 4e–24 e-value, respectively) carry out the same function in *E. coli* and are representative members of the iron–sulfur cluster assembly.

**FIGURE 5:**
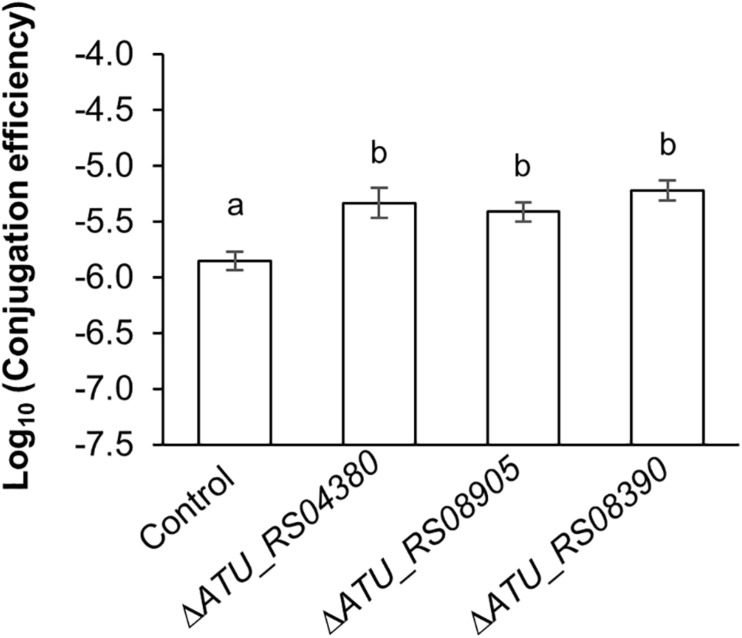
Effect of up-mutant homologs gene-knockout in *A. tumefaciens* on IncP1-type conjugation. The conjugation reaction was performed for 1 h. Data are presented as mean ± standard error of the mean (SEM) within six experimental replicates (*n* = 6). Different letters indicate significant differences between mutants and wild-type control at *p* < 0.05 using Tukey HSD multiple comparison analysis. BY4742 was used as the recipient. C58C1 parental strain was used as the control.

All examined mutants showed significantly higher TKC efficiency compared to the parental strain (Figure 5). The Δ*ATU_RS08390* mutant showed a fourfold increase in TKC efficiency compared to the parental strain. In addition, threefold increases in conjugation efficiency were observed in Δ*ATU_RS04380* and Δ*ATU_RS08905* mutants (Figure 5). These results suggest that these homologous mutant genes (*ATU_RS04380*, *ATU_RS08390*, and *ATU_RS08905*) have similar characteristics in terms of enhancing the TKC efficiency facilitated by IncP1-type T4SS machinery to the recipient cell.

## Discussion

The enhancing effect of the three mutants to both prokaryotes and eukaryotes is probably IncP1-type T4SS-dependent, and it was commonly observed in two donor species belonging to different classes (Figures 1, 4C,D, 5).

**FIGURE 6:**
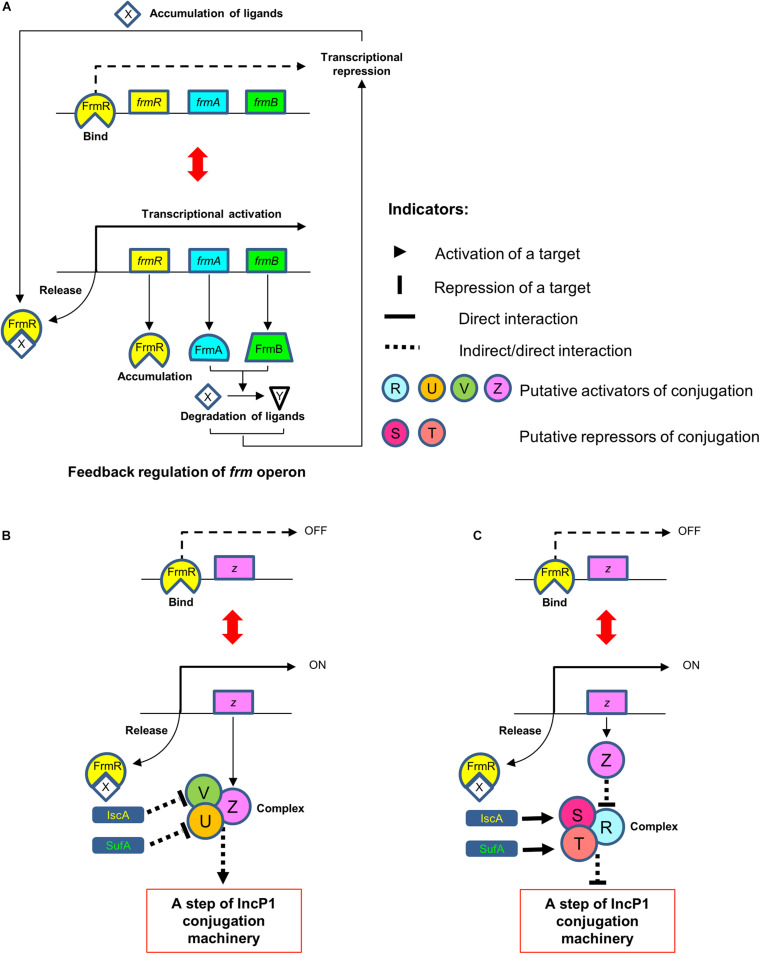
Possible model mechanisms of the FrmR, SufA, and IscA protein interactions within *E. coli* donor in repressing the conjugation of IncP1α plasmid. **(A)** Feedback regulation of *frm* operon. FrmR is a transcriptional repressor of the *frm* operon. The accumulation of ligands (e.g., formaldehyde) causes the inactivation of FrmR repressor activity by binding to the ligands, consequently activates the transcriptional activity of this operon. This transcriptional activation leads to the expression of the downstream genes, *frmA* and *frmB*, which encode FrmA and FrmB for formaldehyde detoxification. **(B,C)** FrmR is also predicted to be a transcriptional repressor on the operon of another target factor (activator) within the *E. coli* donor which represents as factor Z. IscA and SufA are predict to work in repressing the activators (factors V and U, respectively) either by directly or indirectly **(B)** or directly activate the repressors (factors S and T, respectively) **(C)**. At the same time, both of these activators may form a complex with the FrmR target factor (Z) in order to activate conjugation **(B)** or the repressor may form a complex with other factor (factor R) repressed by factor Z either directly or indirectly, resulting to the repression of conjugation **(C)**, either by directly or indirectly. Based on these model mechanisms, the FrmR, SufA, and IscA proteins are possible to repress the conjugation at the identical step(s) of IncP1 conjugation machinery.

FrmR is a formaldehyde-sensing transcriptional repressor of the *frm* operon (Denby et al., 2016; Osman et al., 2016). On the basis of the experimental result shown in Figure 2A, no additional effect on conjugation efficiency was observed in the Δ*frmR* mutant due to the addition of formaldehyde. These results suggested that the absence or inactivation of FrmR from the *frm* operon either due to a gene deletion mutation or the binding of this protein to the excessive formaldehyde, respectively, resulted in an increase in conjugation efficiency. In the case of TKC efficiency by the gene mutation within the *frm* operon (Figure 2B), single-KO of both Δ*frmA* and Δ*frmB* conferred no significant difference compared to Δ*frmR*. This was probably due to the accumulation of endogenous ligands, including formaldehyde, caused by the failure of the detoxification mechanism by FrmA and FrmB within the cells, which inactivated FrmR, leading to the increase in conjugation efficiency (Figure 6A). A previous study showing that the deletion of *frmA* causes increase in basal *frmR* promoter activity as well as improved sensitivity to formaldehyde supports our expectation (Woolston et al., 2018). In the case of the double-KO mutants, Δ*frmA*Δ*frmR* and Δ*frmB*Δ*frmR*, no additional increase in TKC efficiency was observed between these mutants and single-KO mutants. On the basis of this status, we propose that FrmR represses the expression of other target factor(s) within the *E. coli* donor which may increase the conjugation efficiency (Figure 6 and Supplementary Figure 5).

A previous study reported that the autoinducers, *N*-acyl homoserine lactones (AHLs), such as *N*-(3-oxododecanoyl)-_L_-homoserine lactone (OdDHL), produced by *Pseudomonas aeruginosa PAO1*, are capable to repress the conjugation of an IncP1α plasmid (RP4). The repressing mechanism was explained to be caused by a decrease in the expression of the *traI* conjugation-related gene in the RP4 plasmid, which results from transcriptional repression by AHLs-SdiA in donor *E. coli*, by binding to the SdiA-box located at the promoter sequence of *traI* (Lu et al., 2017). However, *E. coli* does not produce AHLs, and no significant effect was observed on conjugation efficiency even though the OdDHL was exogenously supplied in the conjugation reaction mixture (Supplementary Figure 4). On the basis of this status, at least under our experimental conditions, it can be concluded that the increase in conjugation efficiency by the up-mutants is not related to the autoinducer-mediated mechanism.

FrmR belongs to the CsoR/RcnR metal ion-sensing transcriptional repressor family, and the family phylogenetically consists of three clades, namely, CsoRs, RcnRs, and FrmRs (Chen et al., 2016). In *E. coli*, *frmR* and *rcnR* genes are encoded on its genome, and only the *ATU_RS04380* gene, which is in the CsoR clade, has been found in the *A. tumefaciens* C58 genome. This suggests that, although it is possible that CsoR/RcnR family proteins might affect IncP1 plasmid conjugation, the screening and results shown in Figures 1A, 5 indicate that FrmR has specificity for the regulation of IncP1 plasmid conjugation in *E. coli*.

In *E. coli*, the *iscA*, *sufA*, and *erpA* genes are paralogs and coding members of iron–sulfur cluster carrier proteins having overlapping functions (Loiseau et al., 2007; Roche et al., 2013). The KO mutant of *erpA* is not included in the Keio library because of its essentiality. In addition, double-KO of Δ*iscA*Δ*sufA* genes result in synthetic lethality under aerobic condition (Vinella et al., 2009). Thus, this double-KO mutant was excluded from this conjugation assessment, and the KO mutation of either of these genes with Δ*frmR* was constructed to confirm the gene interaction. The conjugation efficiency of double-KO mutants Δ*iscA*Δ*frmR* and Δ*frmR*Δ*sufA* did not exhibit any synergistic increase in conjugation efficiency and was comparable with that of Δ*iscA*, Δ*sufA*, and Δ*frmR* single-KO mutants (Figure 2C). The loss of expression of any of the three up-mutant genes did not lead to attenuated expression of other two genes (Figure 3B). Based on this status, we predicted that FrmR, SufA, and IscA target to different unknown factor(s) (activator or repressor) within the *E. coli* donor cells and independently affect the conjugation mechanism. At this status, we predict that the defect of FrmR, SufA, and IscA probably target to the activator(s) which may direct or indirectly activate the conjugation mechanism. In addition, SufA and IscA are also predicted to work in activating or repressing the conjugation mechanism indirectly, either by repressing or activating respective unknown target factor(s). This prediction was made since no decreasing effect in conjugation efficiency (or at comparable level to that of parental strain) was observed in Δ*sufA* and Δ*iscA* single- as well as Δ*iscA*Δ*frmR* and Δ*frmR*Δ*sufA* double-KO mutants, regardless in the presence of Δ*frmR.* This probably due to the absence of complementation effect between both SufA and IscA. Thus, we predict that both SufA and IscA are probably necessary in activating or repressing the conjugation mechanism indirectly with unknown target factor of FrmR.

These results suggest that the unknown target factors of these three genes form a complex in order to activate or repress the conjugation, either by directly or indirectly at an identical step(s) of IncP1 conjugation machinery although the exact mechanism beyond this phenomenon remains unknown. Since KO mutants for *iscA*, *sufA*, and their agrobacterial homologs have been commonly shown to increase conjugation efficiency (Figures 1, 5), iron–sulfur cluster delivery deficiency probably causes a common physiological status, which specifically promotes the IncP1-type conjugation system (Figures 6B,C and Supplementary Figures 5A,B).

On the basis of the results from single- and double-KO conjugation experiment and the relation with basal gene expression, as well as the known functions of FrmR, SufA, and IscA, we propose models for the repression mechanism of the IncP1-type conjugation system (Figure 6). We deduce that SufA and IscA work in repressing other target factors (activators) within the *E. coli* donor either by directly or indirectly. At the same time, the inactivation of FrmR, which may also be a repressor of other target factor (activator), will derepress the expression of that particular factor. The unknown target factors of FrmR, IscA, and SufA may form a complex to activate the conjugation, either by directly or indirectly at an identical step of IncP1 conjugation machinery (Figure 6B). At present, there are still several models that we deduce how these three gene products interact with other unknown target factors in the process of IncP1α conjugation which fits to our results (Figure 6C and Supplementary Figure 5).

Lastly, IncP1-type T4SS carries high potential for the application of a gene introduction system into various organisms (Dodsworth et al., 2010; Norberg et al., 2011; Moriguchi et al., 2013a). The data regarding the mutants isolated in this study should be an appropriate basis for the breeding of donor strains from various proteobacteria, each of which carries high cytological affinity with target organisms in addition to high conjugation ability. Further characterization in terms of possible gene interaction within the chromosomal mutants based on physiological analysis with the possible regulators within the IncP1 plasmids will lead for better understanding of the isolated genes’ diversity with the TKC mechanism. Additionally, it will be interesting to determine the specificity of T4SS-mediated IncQ conjugal transfer in the isolated mutant strains by using Ti plasmid (VirB/D4-T4SS system) in comparison to IncP1-type system as it has been reported to serve as a delivery system in *Agrobacterium* (Bohne et al., 1998; Ohmine et al., 2018; Kiyokawa et al., 2020).

## Data Availability Statement

The raw data supporting the conclusions of this article will be made available by the authors, without undue reservation, to any qualified researcher.

## Author Contributions

KM, SY, and KS conceived and designed the experiments. FZ, KM, and YC performed the experiments and analyzed the data. SY and KK contributed the reagents and materials. FZ and KM wrote the manuscript. FZ, KM, YC, SY, KK, and KS revised the manuscript. All authors read and approved the manuscript.

## Conflict of Interest

The authors declare that the research was conducted in the absence of any commercial or financial relationships that could be construed as a potential conflict of interest.

## Abbreviations

AHL: acyl homoserine lactones
HGT: horizontal gene transfer
NBRP: National BioResource Project
SD: synthetic defined
SEM: standard error of the mean
TKC: *trans*-kingdom conjugation
TNB: Tris-HCl with NaCl buffer
VGT: vertical gene transfer
YPD: yeast-extract/peptone/dextrose.
