# Supplementary material for: Isolation and Analysis of Donor Chromosomal Genes Whose Deficiency Is Responsible for Accelerating Bacterial and Trans-Kingdom Conjugations by IncP1 T4SS Machinery
Source: Front Microbiol. 2021 May 20;12:620535. doi: 10.3389/fmicb.2021.620535 (PMC8174662; doi:10.3389/fmicb.2021.620535)
Supplement: Supplementary file 1 [file Data_Sheet_1.PDF]

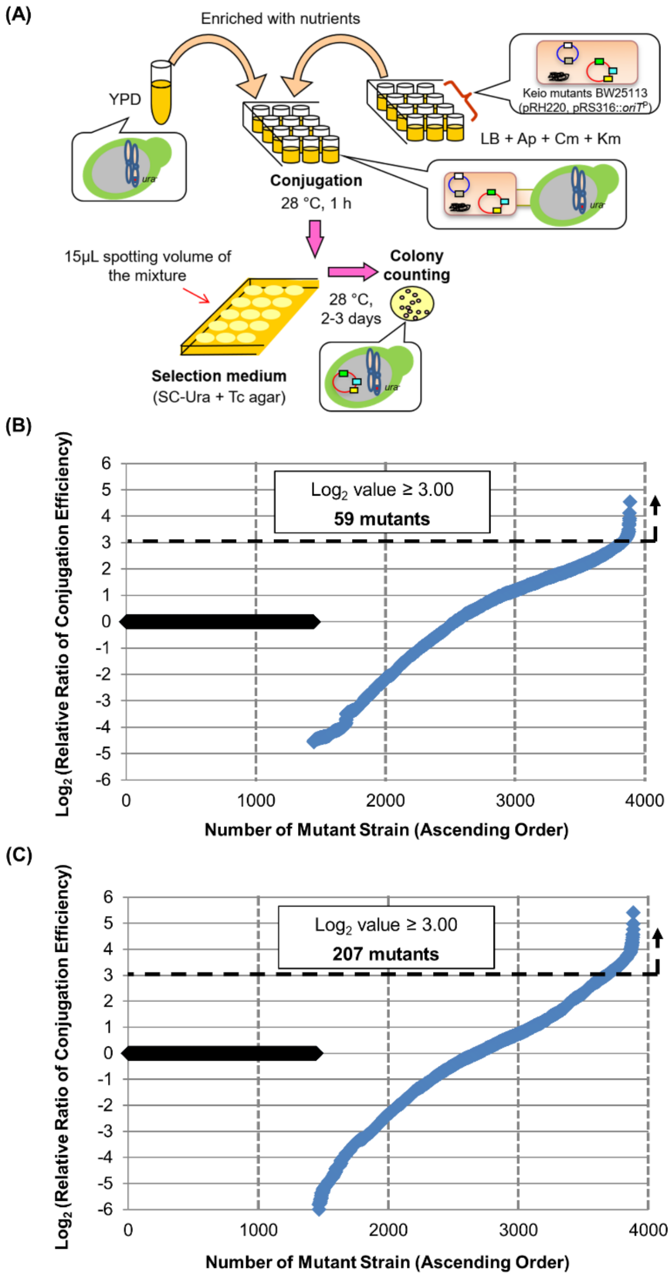

Figure S1: First screening strategy of *E. coli* Keio single-KO mutants (3,884 mutants). **(A)** Overall flowchart of the first screening. **(B)** Distribution pattern of the relative TKC efficiency by the mutant donors in 1-h conjugation reaction normalized using median value of all control reactions used in this screening. **(C)** Distribution pattern of the TKC efficiency by the mutant donors in 1-h conjugation reaction normalized using the median value of control in every experiment. Log_2[number of transconjugants / median transconjugant number of control colonies] values for each mutant are plotted in ascending order. In this screening, 1,446 mutants showed values lower than the detection limit (indicated as black diamonds). Two hundred thirty-three mutants with TKC efficiency of [log_2 value $\geq$ 3] were isolated based on these two different methods of calculations and subjected for the second screening. BY4742 was used as the recipient.

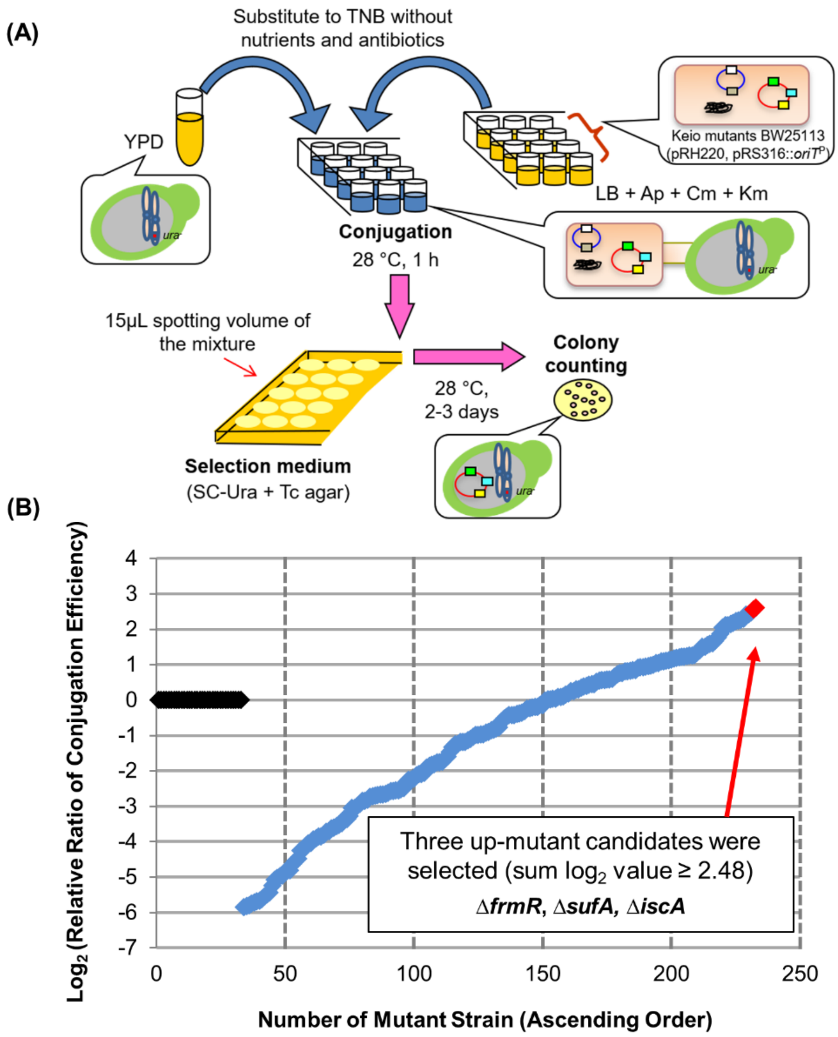

Figure S2: Second screening strategy of *E. coli* Keio single-KO mutants (233 mutants). **(A)** Overall flowchart of the second screening and **(B)** distribution pattern of the relative TKC efficiency within triplicate data (n = 3) by the mutant donors in 1-h conjugation reaction normalized using the median value of control in every experimental replicates. Log_2[number of transconjugants / median transconjugant number of control colonies] values for each mutant are plotted in ascending order. In this screening, 33 mutants showed values lower than the detection limit (indicated as black diamonds). Three mutants with increased conjugation efficiencies within triplicate experiments [sum $\log_2$ value $\geq$ 2.48] were picked up and subjected to characterization analysis. BY4742 was used as the recipient.

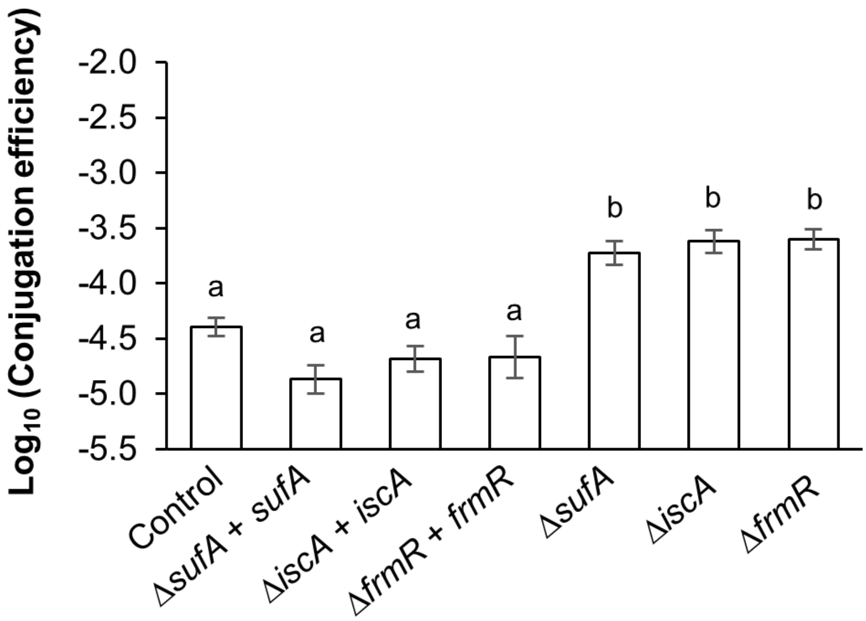

Figure S3: Complementation analysis of *frmR, iscA,* and *sufA* mutants. *E. coli* single-KO mutants (Δ*frmR*, Δ*iscA*, and Δ*sufA*) were transformed with pJP5603sacBGmR (+*frmR* or *iscA* or *sufA* including each adjacent sequences) via conjugation by S17-1 λ*pir*. The primary homologous recombination was then induced within the genome of the Δ*frmR*, Δ*iscA*, and Δ*sufA*, respectively. Regarding the secondary homologous recombination step, pRH220 and pRS316::*oriT*^{P} were introduced into the primary complemented strains, prior to the induction of secondary homologous recombination by culturing the strains on the LB media-containing Ap, Cm and 10% sucrose. The successful complemented strains with the completely removal of kanamycin resistance gene cassette was isolated on LB media-containing Ap and Cm. Assessment on the conjugation efficiency (within 1 h co-cultivation) by these complemented strains in comparison to wild-type and single-KO mutants of Δ*frmR*, Δ*iscA*, and Δ*sufA* was performed. Data are presented as mean ± standard error of mean (SEM) for nine experimental replicates (n = 9) (control, Δ*sufA* + *sufA*, and Δ*frmR* + *frmR*); eight experimental replicates (n = 8) (Δ*frmR,* Δ*iscA,* and Δ*sufA*); six experimental replicates (n = 6) (Δ*iscA* + *iscA*). BY4742 was used as the recipient. Different letters indicate significant differences between mutants and wild-type control at $p < 0.05$ using Tukey HSD multiple comparison analysis. BW25113 parental strain was used as the control.

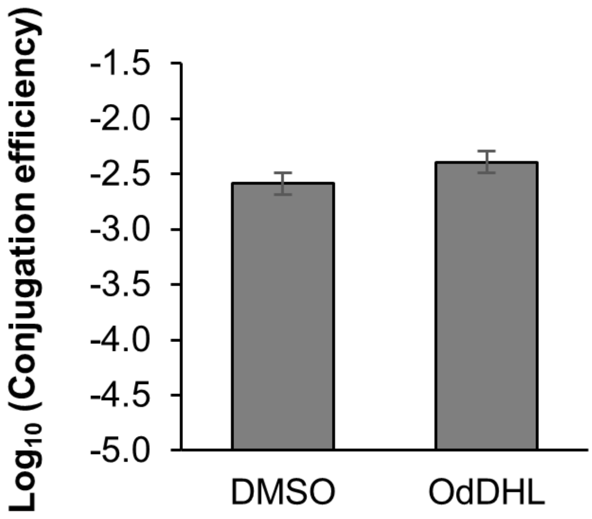

Figure S4: Assessment on the effect of conjugation efficiency of RP4 plasmid from wild-type *E. coli* BW25113 strain in the absence or presence of *N*-(3-oxododecanoyl)-L-homoserine lactone (OdDHL) in a 6 h conjugation reaction. Data are presented as mean ± standard error mean (SEM) for four experimental replicates (n = 4). SY327 was used as the recipient. Asterisk (*) indicates statistically significant differences at $p < 0.05$ (two-tailed *t*-test) compared to control (without exogenously supplied OdDHL) in the conjugation reaction mixture of the corresponding donor *E. coli* strain.

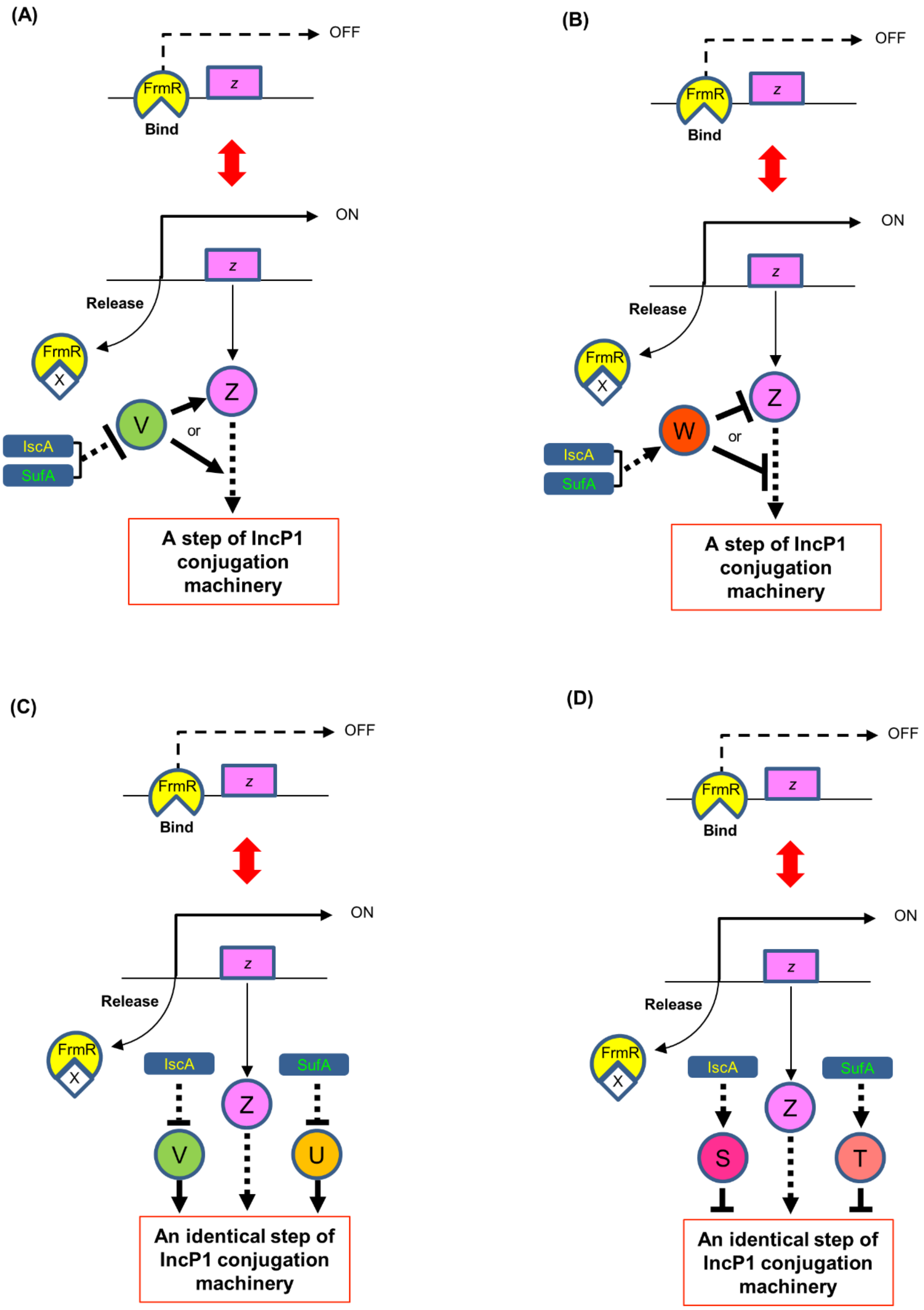

(A)
OFF
FrmR
z
Bind
ON
z
Release
FrmR
X
Z
V
or
IscA
SufA
A step of IncP1 conjugation machinery
(B)
OFF
FrmR
z
Bind
ON
z
Release
FrmR
X
Z
W
or
IscA
SufA
A step of IncP1 conjugation machinery
(C)
OFF
FrmR
z
Bind
ON
z
Release
FrmR
X
IscA
SufA
Z
V
U
An identical step of IncP1 conjugation machinery
(D)
OFF
FrmR
z
Bind
ON
z
Release
FrmR
X
IscA
SufA
Z
S
T
An identical step of IncP1 conjugation machinery

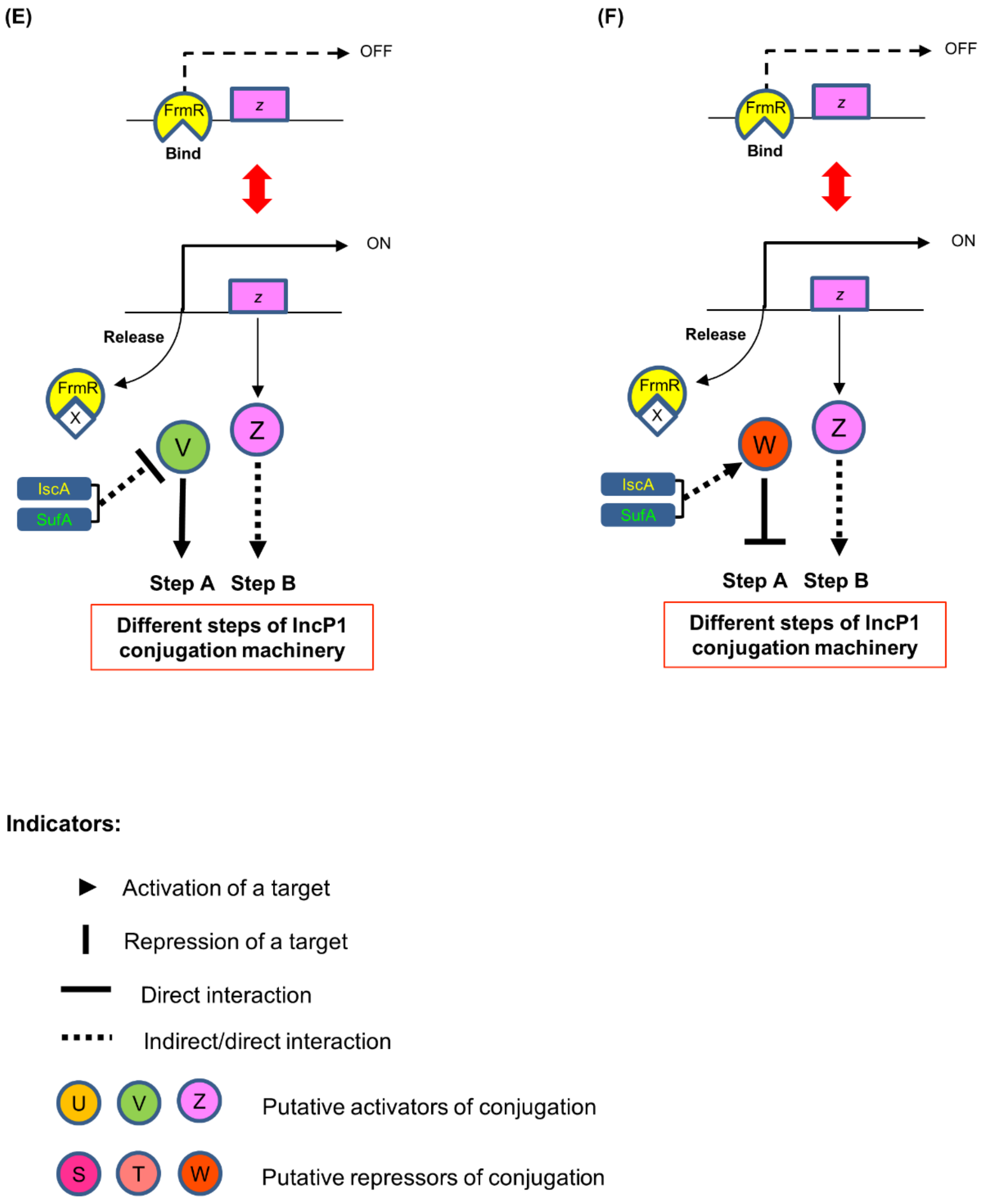

Figure S5: Additional predicted mechanisms for FrmR, IscA, and SufA interactions within *E. coli* donor in repressing the conjugation of IncP1α plasmid. FrmR is also predicted to be a transcriptional repressor on the operon of another target factor (activator) which represent as Z. IscA and SufA are predicted to work cooperatively in repressing an activator (factor V) **(A)** or activating a repressor (factor W) **(B)** either by directly or indirectly. These factors may directly activate or repress the factor Z (or other downstream factor(s) of Z), which consequently activate the conjugation either by directly or indirectly, respectively. Based on these model mechanisms, FrmR, IscA, and SufA are predicted to repress the conjugation at the identical step(s) of IncP1 conjugation

machinery. Besides that, there is also a possibility where both IscA and SufA work uncooperatively in repressing activators (factors V and U) **(C)** or activating repressors (factors S and T) **(D)** either by directly or indirectly, resulting to direct activation or repression of the conjugation, respectively. In parallel, the factor Z also predicted to directly or indirectly activate the conjugation. Based on this status, FrmR, IscA, and SufA are also predicted to repress the conjugation at the identical step(s) of IncP1 conjuagtion machinery. In addition, there is also a possibility where the target factor(s) for the three proteins regulate different steps of IncP1 conjugation machinery. Based on this model, the activator (Z) does not interact to the activator (V) **(E)** or the repressor (W) **(F)**, and activate the conjugation at different step(s) of the conjugation machinery from those of the activator (V) or the repressor (W), which are repressed or activated by IscA and SufA, respectively. Based on this status, FrmR, IscA, and SufA genes are predicted to repress the conjugation at different steps of IncP1 conjugation machinery.
