# Supplementary material for: Isolation and Analysis of Donor Chromosomal Genes Whose Deficiency Is Responsible for Accelerating Bacterial and Trans-Kingdom Conjugations by IncP1 T4SS Machinery
Source: Front Microbiol. 2021 May 20;12:620535. doi: 10.3389/fmicb.2021.620535 (PMC8174662; doi:10.3389/fmicb.2021.620535)
Supplement: Supplementary file 2 [file Data_Sheet_2.PDF]

Table S1: Primers and PCR conditions used in quantitative real-time PCR

Gene	Primer sequence (5′-3′)		Amplicon size	Reference
	Forward	Reverse		
traI	GCTGAAATGCTATTGCCGCG	TATCGAAGCCGTTTAGCCGC	178 bp	This study
traJ	AGGGCTACAAAATCACGGGC	TGCTTCTCTTCGATCTTCGCC	178 bp	This study
traK	TCTCCTACGAGACGTTCCGC	TTGGGTTGAAGGTGAAGCCG	195 bp	This study
trbL	TCGACAACGTATTGCAGCGC	CGGTGAAGATGGTGAACCGC	189 bp	This study
sufA	GGCGATACACATCCGTGAGC	AGCGGGACAAACAGCTTCG	171 bp	This study
iscA	AGAACCTCCGGGTGTTCAGG	AACCCTTCGTTCAGGCCTTC	173 bp	This study
frmR	GGGGCAGATTGATGCTCTGG	AACGGATTGGCTGACTTCGC	181 bp	This study
cysG	CGCAGTCGCTGGCAAACAACGA	TTCAGTGTCAGCAGCCCGGCAT	136 bp	This study
rrsA	CTCTTGCCATCGGATGTGCCCA	CCAGTGTGGCTGGTCATCCTCTCA	105 bp	Zhou et al., 2011

Optimized PCR conditions of FastStart Essential DNA Green Master for the usage in LightCycler® 96 Instrument. Pre-incubation: 95 °C for 10 min, followed by 45 cycles of three-steps amplification: 95 °C for 10 s, 60 °C for 10 s; extension, and 72 °C for 10 s. For melting curve construction, 95 °C for 10 s, 65 °C for 1 min, and 97 °C for 1 s.
