# Supplementary material for: Isolation and Analysis of Donor Chromosomal Genes Whose Deficiency Is Responsible for Accelerating Bacterial and Trans-Kingdom Conjugations by IncP1 T4SS Machinery
Source: Front Microbiol. 2021 May 20;12:620535. doi: 10.3389/fmicb.2021.620535 (PMC8174662; doi:10.3389/fmicb.2021.620535)
Supplement: Supplementary file 3 [file Data_Sheet_3.pdf]

Supplementary Materials and Methods

Construction of *E. coli* Double-Knockout Mutant Strains

All primers used in this experiment are listed in **Supplementary Table 2**. The secondary in-frame gene deletion was performed using the same approach as previously reported by Baba *et al*. (2006). *Km*^{R} gene cassettes were amplified from genomic DNAs of Δ*sufA* and Δ*frmR* strains replaced with *Km*^{R} gene cassette. The cassettes included ≥ 100 bp of upstream and downstream of the deleted gene sequences, by using primer sets: F001-F002 and F003-F004, respectively. The amplified fragments were then introduced into single-knockout mutants without *Km*^{R} gene cassette (Δ*frmR*, Δ*iscA*, and Δ*frmB*) to generate double-knockout mutants: Δ*frmR*Δ*sufA*, Δ*iscA*Δ*frmR*, and Δ*frmB*Δ*frmR* according to the instruction manual of Red/ET Recombination kit purchased from Gene Bridges GmbH (Heidelberg, Germany). Due to an adjoining position of *frmA* and *frmR* genes within *frm* operon, the construction of Δ*frmA*Δ*frmR* double-knockout strain using this strategy was almost impossible. This mutant was constructed by amplifying the fragments between ≥ 100 bp upstream region of Δ*frmR* and the stop codon of *Km*^{R} gene cassette (F003-F006) and between the start codon of *Km*^{R} gene and ≥ 100 bp downstream region of Δ*frmA* (F005-F007). Then, the knockout region of this mutant was generated by using F003-F007 primer pair through the assembly of these two fragments via 2-step and 3-step PCR according to the instruction manual of KOD-Plus-Neo purchased from Toyobo Co., Ltd. (Osaka, Japan). The sequences of Δ*sufA*, Δ*frmR* and Δ*frmA* were obtained from NBRP *E. coli* strains website (https://shigen.nig.ac.jp/ecoli/strain/resource/keioCollection/list/).

Construction of Knockout Mutant Strains for Up-mutant Homologs in *A. tumefaciens*

All primers used in this experiment are listed in **Supplementary Table 2**. In order to construct the *A. tumefaciens* single-knockout mutants, the suicide vector pK18mobsacB in combination with homologous genes (*ATU_RS04380*, NCBI accession number: WP_010971229.1 (NP_353911.2); *ATU_RS08905*, NCBI accession number: WP_010971889.1 (NP_354803.1); *ATU_RS08390*, NCBI accession number: WP_010971818.1 (NP_354701.1), respectively were constructed. These genes showed high homology with *E. coli* up-mutant genes: *sufA*, *iscA*, and *frmR*, based on BlastP search in NCBI database. To perform in-frame target gene deletion, two plasmids were constructed per target gene. For the primary plasmid construction, flanking region of the target gene fragment (approximately 1000-1500 bp) was amplified by PCR for *ATU_RS04380*, *ATU_RS08905*, and *ATU_RS08390* using the primer sets: F008-F009, F010-F011, and F012-F013, respectively. Then, the respective fragment was assembled into pK18mobsacB plasmid in between *BamH*I and *EcoR*I restriction site. Then, the secondary plasmids were constructed by removing out each target gene sequence of *ATU_RS04380*, *ATU_RS08905*, and *ATU_RS08390* using the primer sets: F014-F015, F016-F017, and F018-F019, respectively, by inverse PCR. Each fragment was then

circularized by using NEBuilder® HiFi DNA Assembly Cloning Kit. Construction of *A. tumefaciens* homologous gene deletion mutants (Δ*ATU_RS04380,* Δ*ATU_RS08905*, and Δ*ATU_RS08390*) was performed using the successfully constructed suicide vector plasmids: pK18mobsacBΔ*ATU_RS04380*, pK18mobsacBΔ*ATU_RS08905*, and pK18mobsacBΔ*ATU_RS08390*, respectively. Similar methodology was performed as previously reported study by Schäfer *et al.* (1994).
