# Supplementary material for: Isolation and Analysis of Donor Chromosomal Genes Whose Deficiency Is Responsible for Accelerating Bacterial and Trans-Kingdom Conjugations by IncP1 T4SS Machinery
Source: Front Microbiol. 2021 May 20;12:620535. doi: 10.3389/fmicb.2021.620535 (PMC8174662; doi:10.3389/fmicb.2021.620535)
Supplement: Supplementary file 4 [file Data_Sheet_4.pdf]

Table S2: Primers used for the construction of *E. coli* double-knockout and *A. tumefaciens* up-mutant homologs gene-knockout mutants

Primer name	Primer sequence (5'-3')	Purpose
F001	CGGTAAAGCCCCTGCGTTTG	Double KO construction (Δ*sufA*)
F002	GTGGCTAACTGGGTGAAGAATC	Double KO construction (Δ*sufA*)
F003	GGGGTATGCATTGACATATAG	Double KO construction (Δ*frmR*)
F004	CAACGATTTCCAGCGGTTTAC	Double KO construction (Δ*frmR*)
F005	ATGATTGAACAAGATGGATTGCAC	Double KO construction (Km^R)
F006	TTAGAAGAACTCGTCAAGAAGG	Double KO construction (Km^R)
F007	GTTCCATCAGTCATTATCTCAG	Double KO construction (Δ*frmA*)
F008	CATGATTACGAATTCCGCCGTGGTAGGAGGCAA	pK18mobsacB-*ATU_RS04380* construction
F009	GACTCTAGAGGATCCTTAGGCACTTGCCGGGCTGA	pK18mobsacB-*ATU_RS04380* construction
F010	CATGATTACGAATTCGGCACAGGACGGCGTTTC	pK18mobsacB-*ATU_RS08905* construction
F011	GACTCTAGAGGATCCTCATCCGCGAACGGCAGG	pK18mobsacB-*ATU_RS08905* construction
F012	CATGATTACGAATTCCTATCGCAAATCGGGAGTGTG	pK18mobsacB-*ATU_RS08390* construction
F013	GACTCTAGAGGATCCCAGCCGATCGGACTGAAATC	pK18mobsacB-*ATU_RS08390* construction
F014	ATTTTTAACTGAAGACCTACATCCGGTA	pK18mobsacBΔ*ATU_RS04380* construction
F015	GTCTTCAGTTAAAAATGCTCCATCAGAA	pK18mobsacBΔ*ATU_RS04380* construction
F016	ATAGCTGAGTCATGATCGCAAAGCCCAT	pK18mobsacBΔ*ATU_RS08905* construction
F017	ATCATGACTCAGCTATCGAGGTGCGACG	pK18mobsacBΔ*ATU_RS08905* construction
F018	CTATGATCATGCACGAGCTTTTCGATTTGA	pK18mobsacBΔ*ATU_RS08390* construction
F019	GCTCGTGCATGATCATAGACCAGCGCGC	pK18mobsacBΔ*ATU_RS08390* construction
